# Comprehensive guidance for optimizing the colloidal quantum dot (CQD) Perovskite solar cells: experiment and simulation

**DOI:** 10.1038/s41598-023-43933-x

**Published:** 2023-10-04

**Authors:** Ali Memari, Mohammad Javadian Sarraf, Seyyed Javad Seyyed Mahdavi Chabok, Leili Motevalizadeh

**Affiliations:** 1 Department of Electrical Engineering, Mashhad Branch, Islamic Azad University, Mashhad, Iran; 2 Department of Physics, Mashhad Branch, Islamic Azad University, Mashhad, Iran

**Keywords:** Electrical and electronic engineering, Quantum dots, Solar cells

## Abstract

CsPbI_3_ perovskite quantum dots (CPQDs) have received great attention due to their potential in large-scale applications. Increasing the efficiency of CPQDs solar cells is an important issue that is addressed in this paper. Here, we have simulated a 14.61% colloidal CPQD solar cell with the least fitting parameter that shows the accuracy of the following results. The absorber layer properties are varied and different power conversion efficiency (PCE) is achieved for the new device. The results show that colloidal CsPbI_3_ material properties have a significant effect on the PCE of the device. Finally, the optimized parameters for the absorber layer are listed and the optimum efficiency of 29.88% was achieved for this case. Our results are interesting that help the researchers to work on CsPbI_3_ materials for the achievement of highly efficient, stable, large-scale, and flexible CPQDs solar cells.

## Introduction

Traditional energy generation from fossil fuels has a negative effect on the environment^1^. Using renewable energy sources instead of fossil fuels is an important issue to overcome this problem in worldwide^2,3^. Solar cells are the most important renewable source that can convert sunlight to electricity^4–11^. Silicon solar cell technology is applicable for industrial applications due to long-term stability and high-power conversion efficiency (PCE)^12,13^. High melting point temperature (1400 °C) and needing for expensive instruments for the fabrication of Si solar cells are the biggest challenge for the production of low-cost solar cells^14,15^. Researchers are working on, low-temperature and easy fabrication of third-generation of solar cells. The most known and important of these types of solar cells are perovskites due to their suitable properties and efficiency achievement above 25% in a short time (nearly one decade)^16^. Recently perovskite quantum dots (PQDs) are received great attention^17^. Mainly $a$-CsPbI_3_ material has been used as an absorber layer in PQDs due to its ideal bandgap of 1.73 eV^18,19^. Suitable properties such as narrow photoluminescence (PL), high carrier mobility, tunable absorbance, and great defect tolerance enable low non-radiative recombination despite huge defect density^18^. Good crystallinity and morphological control are not necessary simultaneously for the film deposition of colloidal CsPbI_3_ QDs and this feature makes them suitable for fabrication of large-scale devices via printing techniques^18^. Achievement to the efficiency of 16% for CsPbI_3_ QD solar cells (more than PbS QDs) shows their high potential for next-generation QD solar cells^20^. Hence the focus of this work is the work on simulation of CsPbI_3_ QDs solar cells. Yuan et al.^21^ have fabricated CsPbI_3_ perovskite quantum dot (QD) solar cells by using a series of dopant-free polymeric hole-transporting materials (HTMs) and they have improved charge extraction at QD/polymer interfaces and increased the efficiency to 13%. Mehrabian et al.^22^ have simulated perovskite quantum dot solar cells with three different absorber layers of CsPbI_3_, FAPbI_3_, and CsPbI_3_/ FAPbI_3_, and they optimized their cell by defining the bilayer of the CsPbI_3_/FAPbI_3_ structure by optimization of band gap and they achieved the PCE of 18.55%. In this research, we have simulated the CsPbI_3_ perovskite quantum dot, and compared it to experimental results, at the following, we have investigated the electro-optical properties of CsPbI_3_ CQD on solar cell performance.

## Results and discussion

### Numerical simulation and validation

SCAPS-1D (SCAPS 3306) was used for the simulations. This package solves the Poisson equation (Eq. (1)), and the continuity equation for holes and electrons (Eqs. (2) and (3)) by coupling with Shockley–Read–Hall (SRH) recombination statistics (related to defects in bulk and interfaces) (Eqs. (5) and (6))^23–30^.

1 $$\frac{{d}^{2}}{{dx}^{2}}\Phi \left(x\right)=\frac{q}{{\varepsilon }_{0}{\varepsilon }_{r}}\left(p\left(x\right)-n\left(x\right)+{N}_{D}-{N}_{A}+{n}_{t}^{+}-{n}_{t}^{-}\right)$$

2 $$-\left(\frac{1}{q}\right)\frac{{\partial J}_{p}}{\partial x}-{U}_{p}+G=\frac{\partial p}{\partial t}$$

3 $$\left(\frac{1}{q}\right)\frac{{\partial J}_{n}}{\partial x}-{U}_{n}+G=\frac{\partial n}{\partial t}$$

4 $${U}_{n}={U}_{p}={R}_{Bulk,SRH}+{R}_{Surface, SRH}$$

5 $${R}_{Bulk,SRH}=\frac{np-{n}_{i}^{2}}{{\tau }_{p}\left(n+{n}_{t}\right)+{\tau }_{n}(p+{p}_{t})}$$

6 $${R}_{Surface,SRH}=\frac{np-{n}_{i}^{2}}{\left(n+{n}_{ts}\right)/{S}_{n}+(p+{p}_{t})/{S}_{p}}$$

**Figure 1:**
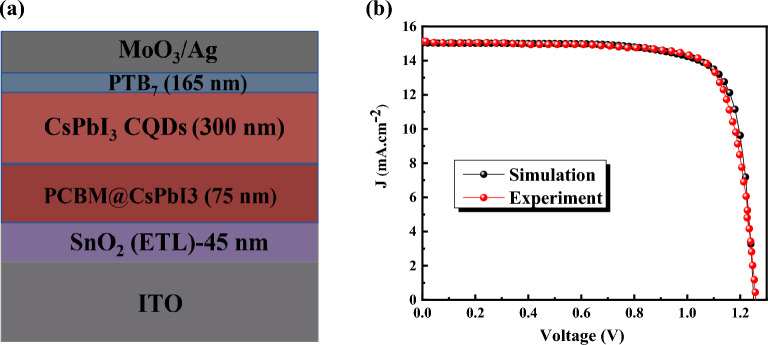
(**a**) Schematic of Perovskite quantum dot solar cells and (**b**) J-V characteristics of Perovskite quantum dot solar cells simulation and experiment.

In Eq. (1), parameter $\Phi$ is the electrostatic potential, *q* is the electrical charge, *ε*_*r*_ and *ε*_*0*_ are the relative and the vacuum permittivity, *p* and *n* are hole and electron concentrations, *N*_*D*_ and *N*_*A*_ are charge impurities of donor and acceptor type, ${n}_{t}^{+}$ and ${n}_{t}^{-}$ are hole and electron trap concentrations, respectively. In Eqs. (2) and (3); *J*_*n*_ and *J*_*p*_ are the electron and hole current densities. *G* is the generation rate. *τ*_*n*_/*τ*_*p*_, *R*_surface_, *S*_*n*_/*S*_*p*_ , *n*_*i*_, *n*_*t*_/*p*_*t*_ , and *n*_*ts*_/*p*_*ts*_ are the electron/hole lifetime, surface recombination rate, surface recombination velocity of the electrons/holes, the intrinsic carrier concentration, the bulk electron/hole concentration of the trap states, and surface trap state concentration, respectively. Figure 1a shows the structure of CsPbI_3_ CQDs. The input data for the simulation is provided in Table 1, and simulation-experiment J-V comparison was shown in Fig. 1b.

**Table 1:** Input electro-optical parameters for the simulation.

Parameter and units	SnO_2_ (ETL)	PCBM@CsPbI_3_ (Buffer)	CsPbI_3_ CQDs (Absorber)	PtB_7_ (HTL)
Thickness (nm)	45 [experiment]	75 [experiment]	300 [experiment]	165 [experiment]
Electron affinity (eV)	4.5 [experiment]	4.25[experiment]	3.99 [experiment]	3.45 [experiment]
Bandgap (eV)	3.6 [experiment]	2 [experiment]	1.76 [experiment]	1.63 [experiment]
Dielectric permittivity (relative)	9	4^31^	6^32^	5^33^
CB effective density of states (cm^-3^)	2.2 × 10^18^ [Ref.^32^]	1.0 × 10^21^^31^	1.1 × 10^20^^32^	1.1 × 10^20^ [fitting]
VB effective density of states (cm^-3^)	1.8 × 10^19^ [Ref.^32^]	2 × 10^20^^31^	8 × 10^19^^32^	8 × 10^19^ [fitting]
Electron mobility (cm^2^V^-1^s^-1^)	100 [Ref.^32^]	1 × 10^−2^^31^	1.6 × 10^1^^32^	1 × 10^−2^^33^
Hole mobility (cm^2^V^-1^s^-1^)	25 [Ref.^32^]	1 × 10^−2^^31^	1.6 × 10^1^^32^	1 × 10^−2^^33^
Shallow uniform donor density N_D_ (cm^-3^)	1.0 × 10^20^ [Ref.^32^]	1.0 × 10^20^^31^	0^32^	0^33^
Shallow uniform acceptor density NA (cm^-3^)	0 [Ref.^32^]	0^31^	1.0 × 10^15^^32^	7.0 × 10^16^^33^
Absorption constant A (cm^−1^ eV^-(½)^)	file	1.0 × 10^5^	5.0 × 10^5^	1.0 × 10^5^
Absorption constant B (eV^(½)^.cm^-1^)	0	0	0	0
Electron thermal velocity (cm/s)	1.0 × 10^7^ [Ref.^32^]	1.0 × 10^7^	1.0 × 10^7^	1.0 × 10^7^
Hole thermal velocity (cm/s)	1.0 × 10^7^ [Ref.^32^]	1.0 × 10^7^	1.0 × 10^7^	1.0 × 10^7^
Electron Life Time (ns)	1.7 × 10^−4^ [fitting]	1 × 10^−2^ [fitting]	2000 [fitting]	14 [fitting]
Hole Life Time (ns)	1.7 × 10^−2^ [fitting]	1 × 10^−2^ [fitting]	2000 [fitting]	14 [fitting]
Electron capture cross section (cm^-2^)	1.0 × 10^−13^ [fitting]	1.0 × 10^−15^ [fitting]	1.0 × 10^–15^ [fitting]	1.0 × 10^−15^ [fitting]
Hole capture cross section (cm^-2^)	1.0 × 10^−15^ [fitting]	1.0 × 10^−15^ [fitting]	1.0 × 10^–15^ [fitting]	1.0 × 10^−15^ [fitting]
Defect density (cm^−3^)	6.0 × 10^18^ [fitting]	1.0 × 10^19^ [fitting]	5.0 × 10^13^ [fitting]	7.0 × 10^15^ [fitting]
Defect energy with respect to valence band energy Ev/type (eV)	+ 1.2(Single acceptor/Gauss)	+ 0.6/Neutral	+ 0.6/Neutral	+ 0.6/Neutral

Data were extracted from valid references, experiment basic, and fittings.

The interfaces properties data are available in SI.

### Experiment details

The SnO_2_ nanoparticle solution was deposited on ITO with spin-coating method at 120 °C for 30 min. PCBM CB solution (5 mg/mL) was spin-coated with speed of 5000 rpm for 30 s. In the following, a dissolved solution of 1.2 M CsI, 0.6 M PbI_2_, and 0.6 M PbBr_2_ in DMSO was used for precursor solution, and then spin-coated at 1500 rpm and 4500 rpm for 15 s and 45 s, respectively. As an HTL layer, PTB_7_ solution (10 mg/mL in chlorobenzene) was spin-coated on CSPbI_3_ CQDs. Finally, MoO_3_ (10 nm) and Ag (nm) electrodes were deposited via thermal evaporation^34^.

## Tuning properties of CsPbI_3_ CQDs (absorber layer)

### Effect of band gap

**Figure 2:**
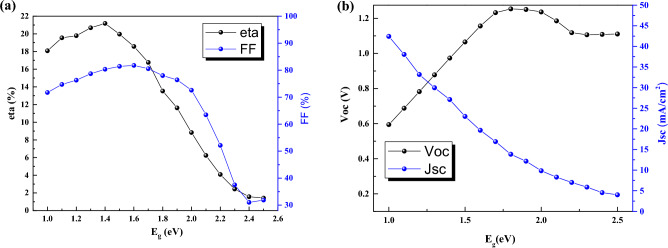
Effect of band gap of CsPbI_3_ CQDs on J-V parameters.

Here, we have studied the effect of band gap of CsPbI_3_ CQDs. By increasing the band gap to value of 1.4 eV, the efficiency will increase to 21%. While, for band gap more than 1.4 eV, V_oc_ is increased to 1.2 V but the efficiency and J_sc_ is decreased and continued to reach 1%, and 3 mA/cm^2^, respectively, for band gap of 2.5 eV (Fig. 2).

**Figure 3:**
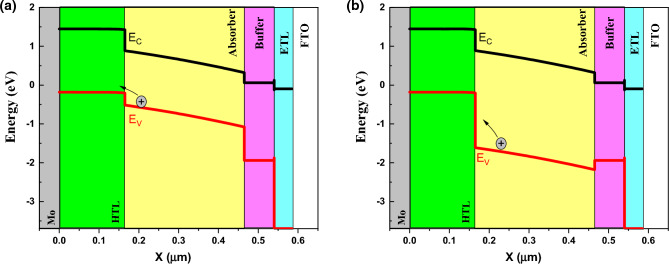
Band structure for band gap of (**a**) 1.4 eV, and (**b**) 2.5 eV. Graphs taken at V = 0 V at light condition.

**Figure 4:**
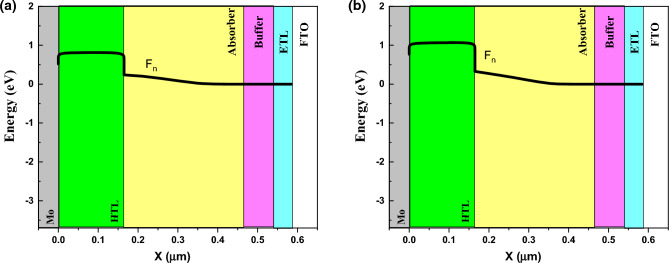
Electron Fermi level for band gap of (**a**) 1.4 eV, and (**b**) 2.5 eV. Graphs taken at V = 0 V at light condition.

By varying the band gap, absorption coefficient also varied due to equation $(A+B/hv)\sqrt{(Eg-hv)}$. For understanding the behavior of band gap effect on performance, we plotted the band structure and electron Fermi level (Figs. 3 and 4) for two cases of high (E_g_ = 1.4 eV) and low efficiency (E_g_ = 2.5 eV). The valence band offset (VBO) between absorber and HTL is increased for the case of E_g_ = 1.5 eV that create a spike against hole transport (Fig. 3) and increase the recombination and leads to J_sc_, FF, and efficiency decrement. The higher V_oc_ for the case of E_g_ = 1.5 eV, is due to electron fermi level increment.

### Effect of electron affinity

**Figure 5:**
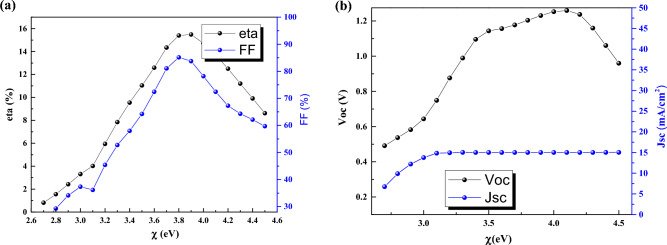
Effect of electron affinity of CsPbI_3_ CQDs on J-V parameters.

Here, we have modified the electron affinity of absorber layer from 2.7 to 4.5 eV (Fig. 5).

**Figure 6:**
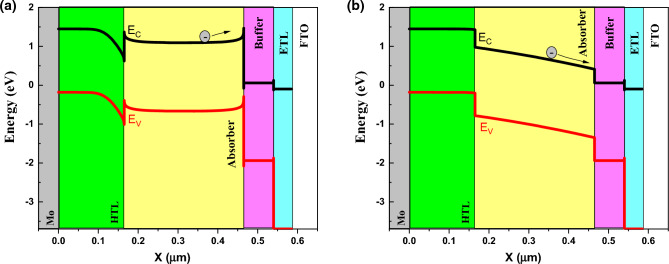
Band structure for electron affinity of (**a**) 2.7 eV, and (**b**) 3.9 eV. Graphs taken at V = 0 V at light condition.

In electron affinity of 2.7 eV and 3.9 eV we have the lowest (0.81%) and highest efficiency (15.48%), respectively. By investigation of band structure, we found that the conduction band offset (CBO) between absorber and buffer in case of 3.9 eV, is in cliff form that help the electrons move easily and speedy (Fig. 6). For the case of 2.7 eV, the electrons in conduction band see a barrier and they are stuck which result in high recombination.

### Effect of electron–hole mobility

**Figure 7:**
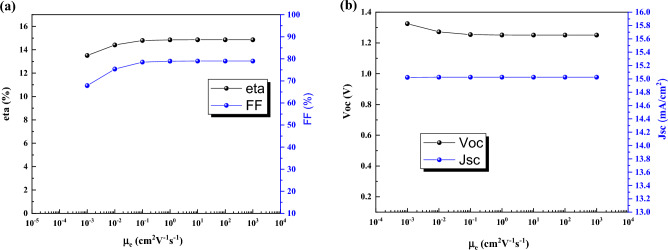
Effect of electron mobility of CsPbI_3_ CQDs on J-V parameters.

**Figure 8:**
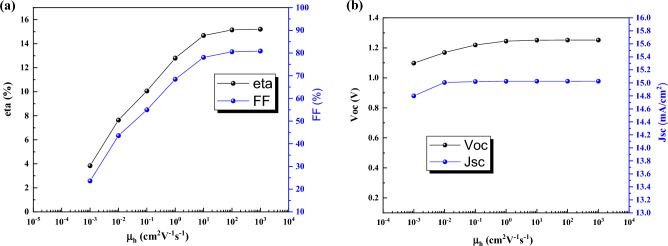
Effect of hole mobility of CsPbI_3_ CQDs on J-V parameters.

Here, the effect of electron and hole mobility on performance are investigated. Electron mobility has low effect on performance (Fig. 7) and it because of the impurity of absorber layer that in p-type semiconductor hole mobility is important. Here, hole mobility has significant impact on J-V characteristics (Fig. 8). By increasing the hole mobility from 10^−3^ cm^2^V^−1^s^−1^ to 10^3^ cm^2^V^−1^s^−1^ the efficiency is increased from 3.84% to 15.20%.

**Figure 9:**
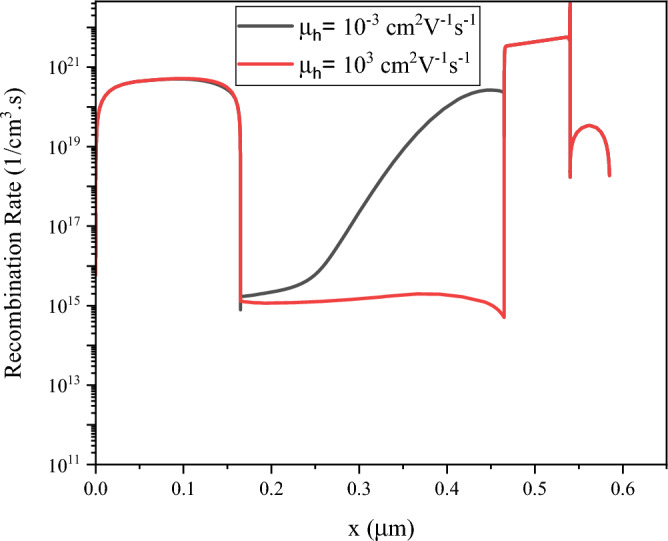
Recombination rate for the hole mobility of (**a**) 10^−3^ cm^2^V^−1^s^−1^ and (**b**) 10^3^ cm^2^V^−1^s^−1^. Graphs taken at V = 0 V at light condition.

The recombination rate for the hole mobility of 10^−3^ cm^2^V^−1^s^−1^ and 10^3^ cm^2^V^−1^s^−1^ is plotted in Fig. 9.

For low values of hole mobility, the recombination rate in perovskite layer is increased severely. Here, for fabrication of QD solar cells with high efficiency we need an absorber layer with high mobility.

### Effect of acceptor density

**Figure 10:**
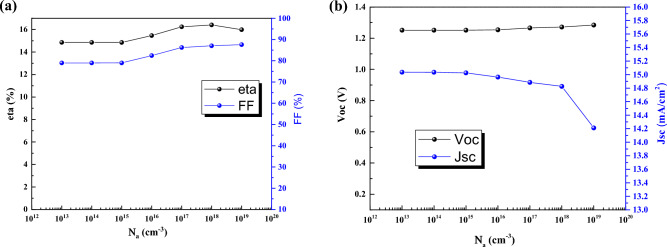
Effect of acceptor density of the absorber of CsPbI_3_ CQDs on J–V parameters.

We modified the doping of absorber layer, acceptor density, by increasing the density of absorber, efficiency, FF and V_oc_ are increased but J_sc_ decreases (Fig. 10).

**Figure 11:**
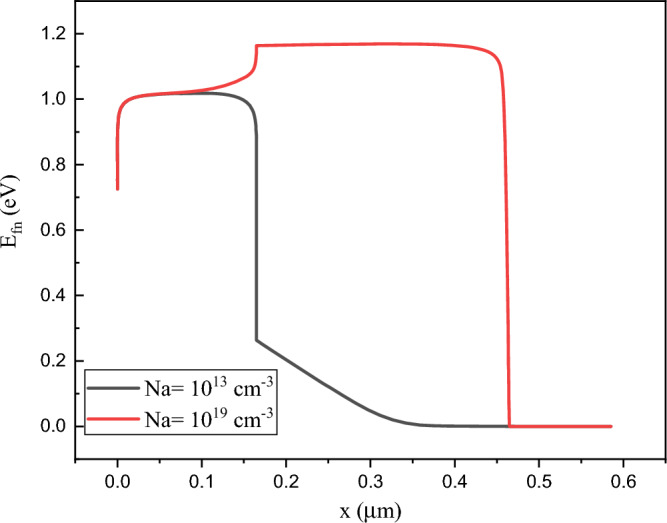
Electron Fermi level for Na = (**a**) 10^13^ cm^−3^, and (**b**) 10^19^ cm^−3^. Graphs taken at V = 0 V at light condition.

As can be seen in Fig. 11, level of electron fermi is enhanced due to higher doping density that helps the electric field in the absorber layer and efficiency increment.

### Effect of defect density

**Figure 12:**
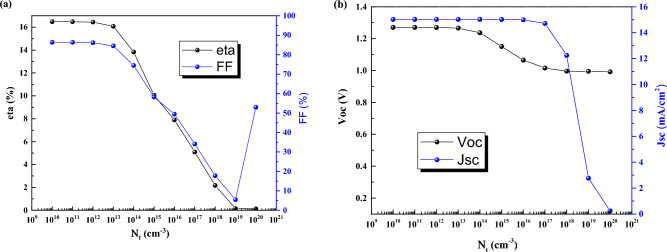
Effect of defect density of the absorber of CsPbI_3_ CQDs on J–V parameters.

The defects formation are the most important factors to have high or low efficiency. Here, we have changed defects density from the low (10^10^ cm^−3^) to high values (10^20^ cm^−3^) (Fig. 12). The high population of defects are detrimental for the device that can reduce the efficiency from 0.12% to 16.49%. It means the high defect density can cause the solar cell device does not work efficiently.

**Figure 13:**
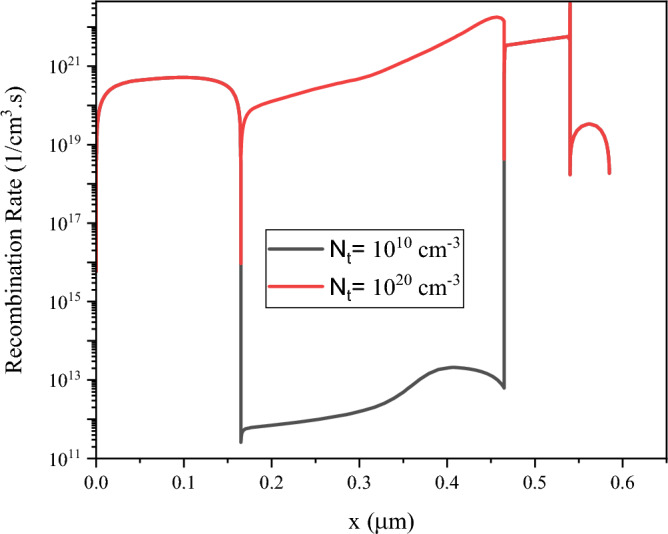
Recombination rate for the defect density of (**a**) 10^10^ cm^−3^ and (**b**) 10^20^ cm^−3^. Graphs taken at V = 0 V at light condition.

The higher defect density will increase the recombination rate at perovskite layer (Fig. 13).

### Effect of defect energy and types

**Figure 14:**
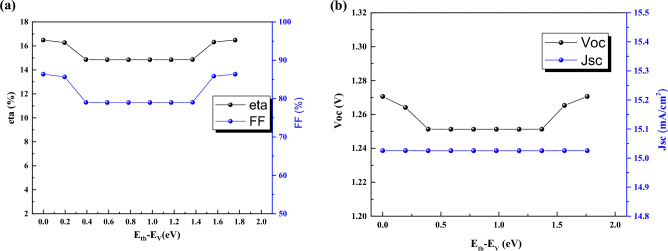
Effect of defect energy of the CsPbI_3_ CQDs on J–V parameters.

Defect energy is important parameter which shows the types of defects in the QD perovskite layer. The defects with energy between 0.4 eV to 1.4 eV are harmful for device due to poor efficiency (Fig. 14).

This behavior is because of recombination rate enhancement.

For defect types we have consider 3 cases,

I. $${\sigma }_{n}={\sigma }_{p}$$
  ${\sigma }_{n}={10}^{-15}$ cm^2^, ${\sigma }_{p}={10}^{-15}$ cm^2^, and N_t_ = 10^15^ cm^−3^ are considered for the below figures.
II. $${\sigma }_{n}>{\sigma }_{p}$$
  ${\sigma }_{n}={10}^{-14}$ cm^2^, ${\sigma }_{p}={10}^{-15}$ cm^2^, and N_t_ = 10^15^ cm^−3^ are considered for the below figures.
III. $${\sigma }_{n}<{\sigma }_{p}$$
  ${\sigma }_{n}={10}^{-15}$ cm^2^, ${\sigma }_{p}={10}^{-14}$ cm^2^, and N_t_ = 10^15^ cm^−3^ are considered for the below figures.

**Figure 15:**
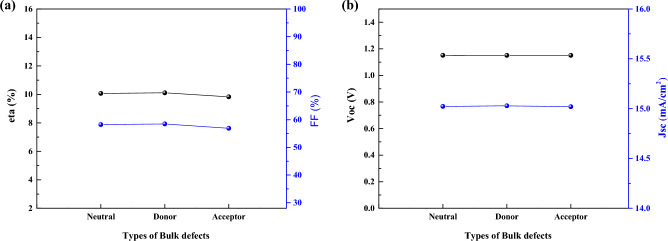
Effect of defect types of the CsPbI_3_ CQDs on J–V parameters.

**Figure 16:**
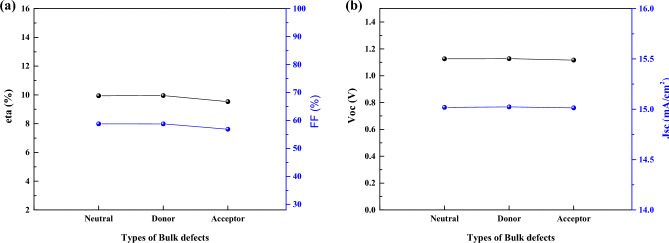
Effect of defect types of the CsPbI_3_ CQDs on J–V parameters.

**Figure 17:**
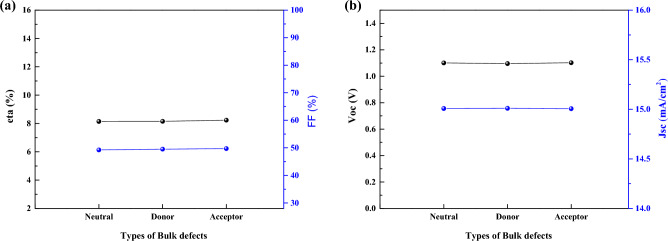
Effect of defect types of the CsPbI_3_ CQDs on J–V parameters.

As we see in 3 cases of I, II, and III (Figs. 15, 16, and 17), there is no significant effect on J-V parameters on 3 types of defects. However, in real situation it is important due to different density of defect (also we show this in previous section), but here we assume the same density for 3 cases of defect type.

### Effect of thickness

**Figure 18:**
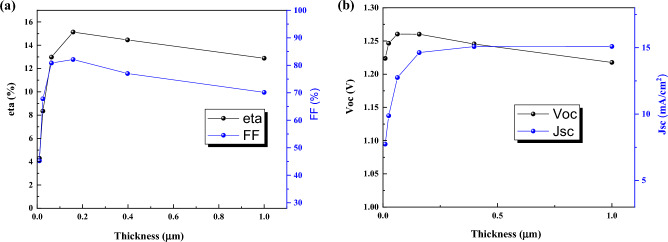
Effect of thicknes of CsPbI_3_ CQDs on J–V parameters.

The effect of thickness is investigated on J-V characteristics (Fig. 18). By increasing the thickness from 10 to 200 nm, the efficiency is enhanced drastically, and for the thickness higher than 200 nm, the efficiency will drop slowly. The thickness between 100 and 350 nm seems suitable for choosing CPQDs solar cells.

## Optimized case

**Figure 19:**
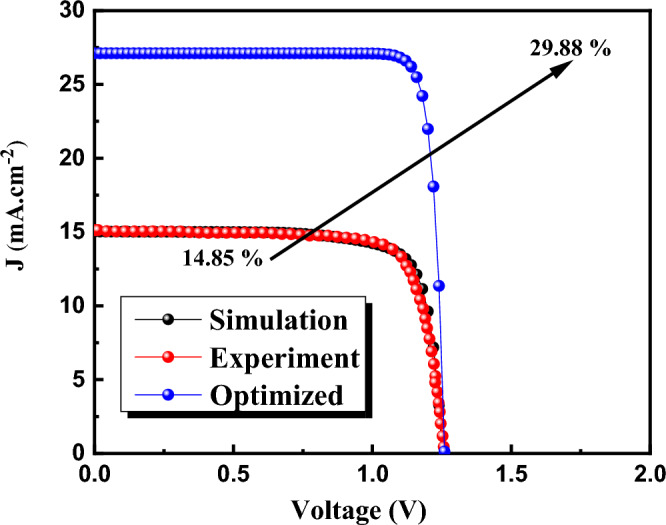
The J-V characteristics comparison between optimized, simulation and experiment case.

Finally, we optimized the cell by definition of new ETL, HTL and optimum electro-optical parameters for HTL/absorber, Buffer/absorber and absorber layer. The efficiency is enhanced from 14.85% to 29.88% (Fig. 19). This improvement eager the researcher to work on CQD Perovskite solar cell for industrial and marketing application in near future.

## Conclusion

This study addressed the comprehensive investigation of absorber layer properties in CsPbI_3_ perovskite quantum dots (CPQDs) solar cells. For the achievement of highly efficient CPQDs solar cells, it is important to choose absorber layer materials with a band gap of 1 − 1.7 eV, an electron affinity of 3.7–4 eV, and electron mobility (${\mu }_{e}$) of 10^−2^–10^3^ cm^2^/V.s, hole mobility (${\mu }_{h}$) of 10^1^–10^3^ cm^2^/V.s, acceptor density (N_a_) of 10^13^–10^19^ cm^-3^, defect properties such as defect density of 10^10^–10^13^ cm^−3^, and Defect energy of 0 − 1.76 eV. For modifying the CsPbI_3_ properties, the material ratio, deposition processes such as temperature and time of deposition, different treatments, adding nanoparticles such as graphene, and using stacked deposition with different sandwich materials are the ways that we have proposed for the modification. The optimum efficiency of 29.88% was achieved for the CPQD solar cell in this research. These results are very promising and eager the researcher work on our proposed model to achieve highly efficient CPQD solar cells.

## Data Availability

The data that support the findings of this study are available from the corresponding author, [MJ.S.], upon reasonable request.
